# Information Is Where You Find It: Perception as an Ecologically Well-Posed Problem

**DOI:** 10.1177/20416695211000366

**Published:** 2021-03-22

**Authors:** William H. Warren

**Affiliations:** Brown University, Providence, Rhode Island, United States

**Keywords:** three-dimensional perception, affordances, higher order motion, locomotion, optic flow, perception/action, sensory ecology

## Abstract

Texts on visual perception typically begin with the following premise: Vision is an ill-posed problem, and perception is underdetermined by the available information. If this were really the case, however, it is hard to see how vision could ever get off the ground. James Gibson’s signal contribution was his hypothesis that *for every perceivable property of the environment, however subtle, there must be a higher order variable of information, however complex, that specifies it*—if only we are clever enough to find them. Such variables are informative about behaviorally relevant properties within the physical and ecological constraints of a species’ niche. *Sensory ecology* is replete with instructive examples, including weakly electric fish, the narwal’s tusk, and insect flight control. In particular, I elaborate the case of passing through gaps. *Optic flow* is sufficient to control locomotion around obstacles and through openings. The *affordances* of the environment, such as gap passability, are specified by action-scaled information. Logically ill-posed problems may thus, on closer inspection, be ecologically well-posed.

## Introduction: Perception as an Ill-Posed Problem

Pick up almost any text on visual perception and you will find the foundational premise that vision is an ill-posed problem. This line of reasoning should be familiar to most readers of this journal. The modern version runs something like this:

The problem of visual perception is an *inverse problem* of recovering a physical scene from an image thereof. The *forward problem* is the problem of image formation, projecting a three-dimensional physical scene onto a two-dimensional image, which is essentially solved. Information is necessarily lost in the course of image formation, most obviously information about the third dimension (and hence size, shape, hidden surfaces, etc.), but so is information about other physical properties (illumination, reflectance, materials, rigidity, mass, and so on). The image is thus inherently *ambiguous*, for the same image could be generated by an infinity of possible scenes (e.g. the Ames room, metamers, foam-rubber rocks, and so on). Therefore, the inverse problem is *ill-posed*, in the formal sense that it does not have a unique solution. Perception is thus *underdetermined* by the available image information. As a consequence, *prior knowledge* is required to *interpret* the image by *inferring* the scene that is most likely to produce it.

This logic is often extended to other perceptual systems as well (e.g., Lewicki et al., 2014).

The trouble with accepting the premise as the starting point for vision science is that it dooms the enterprise before we even get started. If the input for vision was as fundamentally ambiguous as claimed, it is hard to see how a visual system could ever get off the ground. It turns the problem of perception into the problem of prior knowledge: how did the perceptual system acquire, in some extra-sensory manner, the knowledge that is not only a prerequisite for seeing but also a prerequisite for vision to evolve? As Palmer (1999, p. 23) observed, were it not for the fact that our visual systems somehow manage to solve the problem, it would be tempting to conclude that three-dimensional perception is simply impossible.

## Gibson’s Information Hypothesis

James Gibson did not believe this is the way our visual systems solve the problem. As he put it 40 years ago, “Knowledge of the world cannot be explained by supposing that knowledge of the world already exists” (Gibson, 1979, p. 253). The standard line of reasoning is patently circular. Even though it might be logically correct, it is the wrong way to frame the problem of perception: The function of vision is not to solve the inverse problem and reconstruct a veridical description of the physical world (Warren, 2012).

Gibson’s fundamental contribution to the field was his project of *naturalizing* perception, to understand it as a biological, rather than logical, function that evolved to guide adaptive behavior in natural environments. On his view, the function of vision is to keep perceivers *in contact with* behaviorally relevant properties of the world they inhabit, their ecological niche. For visual perception to be possible, there must be sufficient information available to specify such properties, by virtue of physical and ecological laws. This led Gibson to what I will call his Information Hypothesis.

**The Information Hypothesis:** For every perceivable property of the environment, however subtle, there must be a higher order variable of information, however complex, that specifies it.^1^

Note what the hypothesis does *not* say. It does not claim that all properties of the physical world can be veridically perceived. Nor does it claim that all environmental properties are specified by information. In particular, vision scientists cannot assume that the properties defined by classical physics and geometry are relevant descriptors for biological vision. Rather, the claim is that, for environmental properties that *are* successfully perceived, there must be information specific to them—if only we scientists are clever enough and dogged enough to find it. Hence, Gibson’s (1979) call for an *ecological physics* to identify the relevant environmental properties, and an *ecological optics* (*acoustics*, *haptics*, etc.) to analyze the variables of information.

At the core of Gibson’s theory is his concept of information, refined over the course of three decades. He reserved the term for *higher order, spatio-temporal patterns of stimulation* (Gibson, 1950) that are *specific to* environmental properties *important to the animal* (Gibson, 1959). *Specific to* means the patterns are *univocally related* to environmental properties (one-to-one or many-to-one; Gibson, 1959, 1966) by virtue of physical and ecological *laws* (Gibson, 1966, 1979). Specificity is what distinguishes information from probabilistic cues and its significance is illustrated by a simple demonstration. Years ago, in a student project, Michael Kalish trained a connectionist network to catch a ball and manipulated the correlation between the model’s visual input and the ball’s arrival point. The number of learning iterations began to drop when the correlation was >0.90 and plummeted when it was >0.95. A simple neural network can thus rapidly converge on an informational variable when it is specific to a behaviorally relevant property.

Patterns of stimulation are *informative* because they are lawfully generated by, and uniquely specify, an environmental property within the physical and ecological constraints of a species’ niche. Runeson (1988, p. 299) called such nomic constraints “grantors” of information, facts of nature that render patterns of stimulation informative about certain environmental properties. Thanks to gravity, for example, terrestrial animals and objects rest on the ground, making the horizon ratio informative about size (see Equation 3). Physical processes produce regularly textured surfaces, making optical texture gradients informative about slant and shape. There is work to be done to formally develop this ecological concept of information (for differing views, see Jacobs & Michaels, 2007; Lappin, 2016; Withagen & Chemero, 2009), but for now, we can hazard a definition:

**Gibson Information:** Higher order, spatio-temporal variables of stimulation that are specific to behaviorally relevant properties of the environment within the nomic constraints of an ecological niche.

To the extent that this relation holds, logically ill-posed problems become ecologically well-posed. (“Hold on a minute,” I can hear the reader cry, “ecological constraints are just the same as priors!” That reader might wish to make a detour to the sidebar at the end of the article.) The bottom line for perception is whether specific information is available within the constraints of a particular ecological niche. Thus, when faced with a case of successful perceiving or efficacious acting, the Gibsonian refrain is: *What is the information?*

## Information Is Where You Find It

I stole my title from an article by David Dusenbery (1996), who wrote the founding text of the field of sensory ecology (Dusenbery, 1992). In his 1996 paper, he describes how root-knot nematodes exploit thermotaxis to converge on the depth of the root layer, in spite of circadian fluctuations in soil temperature. His take-away:

All organisms extract useful information in their environment, sometimes from surprisingly complex stimulus patterns. (p. 121)

That sounds a lot like Gibson information: complex patterns of stimulation that are informative within an ecological niche. And once you start looking for it, Gibson information is everywhere, in all kinds of energy arrays. Let’s consider a few cases in point.

### Electrolocation in Weakly Electric Fish

**Figure 1.**
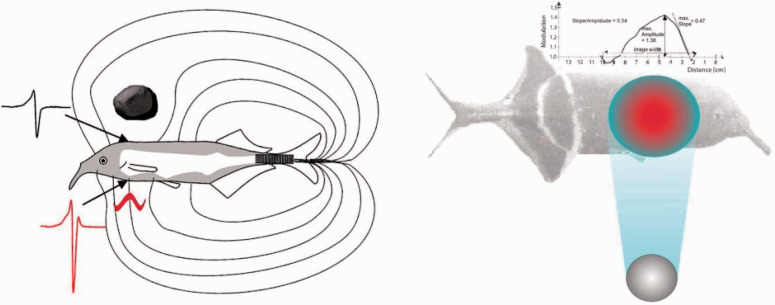
Sketch of electrolocation in the weakly electric fish, *G. petersii*. A: Electric field generated by EOD; field lines illustrate current flow. A resistive rock (above) reduces current density and the amplitude of the local EOD waveform (black). A conductive larva (below) increases current density and its capacitance distorts the local waveform (red). B: Electric “shadow” of a metal sphere cast on skin (red indicates increase and blue decrease in relative amplitude). Above, Mexican hat profile of local voltages measured along the body, illustrating the rostral slope/peak amplitude ratio (from von der Emde, 2006, with permission).

One energy array that has been hijacked as a medium of information is the electric field. Weakly electric fish, which evolved independently in Africa and South America, emit electric organ discharges (EOD) not to stun their prey but to sense their surroundings via *active electrolocation*. The well-studied African mormyrid *Gnathonemus petersii* has a cluster of electrocytes (modified muscle cells) in its tail that generates brief EOD pulses with an amplitude <1 V, creating an electric field in the water around the fish’s body. Thousands of electroreceptor organs along the dorsal and ventral surfaces register the spatial pattern of voltages across the skin, with a higher density near the head. Distortions of the electric field produced by objects alter the voltage pattern (Figure 1A), enabling mormyrids to sense objects up to 12 cm away, localize prey, and orient to their surroundings.

An object within a mormyrid’s field casts an electric “shadow” on its skin, modulating the voltage amplitude with a Mexican-hat profile along the fish’s body (Figure 1B). Objects that are more conductive than water (larvae, plants, and metal) concentrate the current flow (Figure 1A), increasing the amplitude of the profile, whereas resistive objects (rock, clay, dead wood, and plastic) reduce its density, forming an inverted Mexican hat. The location of the shadow’s peak amplitude (max or min) on the skin specifies the bearing direction of the object. So far, so good. The trouble is that the diameter of the shadow increases with both object distance and object size. To make matters worse, the shadow’s peak amplitude decreases with object distance, increases with object size, and varies with its material composition. Electrolocation appears to be—you guessed it—just another ill-posed problem (Lewicki et al., 2014).

But, the Gibsonian persists, *what is the information?* Fortunately, von der Emde (1999, 2006) and his colleagues kept looking. First, consider object distance. They identified a higher order variable that is inversely proportional to distance: the shadow’s *slope/amplitude ratio* (Figure 1B, top). Better yet, this ratio is invariant over changes in the size, shape, and material of most objects. Intuitively, the slope corresponds to the fuzziness of the shadow’s “penumbra,” measured as the maximum slope (*s*) of the Mexican hat function on the rostral side, and the peak amplitude (*a*) corresponds to the center of the shadow’s “umbra.” Object distance is thus specified by the inverse of the slope/amplitude ratio, $D\propto \frac{a}{s}$. When von der Emde trained mormyrids to swim toward the nearer of two objects, the slope/amplitude ratios closely predicted their response functions (von der Emde et al., 1998; Schwarz & von der Emde, 2001).

The authors noticed an interesting exception, however: The slope/amplitude ratio of a perfect metal sphere is shallower than that of other objects, indicating that the sphere is farther away than it actually is. Thus, a metal sphere at 3 cm is a metamer of a metal or plastic cube at 4.5 cm. And indeed, when a metal sphere was compared with metal or plastic cubes of varying sizes, the fishometric distance function shifted by the predicted 1.5 cm. Fortunately for *G. petersii*, perfect metal spheres are a rarity in its niche. This exception proves the rule that the slope/amplitude ratio of the electric shadow is effective information for distance—within ecological constraints.

Building on this distance invariant, higher order information for other object properties can also be derived (von der Emde, 2006). Take object size. The diameter of the electric shadow increases with the size of an object, controlling for its distance. It follows that object size (*S*) is directly proportional to shadow diameter (*d*) divided by the distance due to the slope/amplitude ratio: *S* $\propto \frac{ds}{a}$ .

As mentioned earlier, important families of objects can be identified by their electrical properties. Different materials vary in their *resistance*, whereas only living things have *capacitance*—including insect larvae, the mormyrid’s prey. Resistance and capacitance can be independently sensed by mormyrids, implying that they are also independently specified (Budelli & Caputi, 2000; von der Emde & Ronacher, 1994). Consider resistance. The peak amplitude of the electric shadow decreases with the resistance of an object, controlling for its distance and size. It follows that resistance (*R*) is proportional to an even higher order variable, the inverse of peak amplitude (1/*a*) multiplied by the *S*/*D* given by the preceding variables. Capacitance is specified by the degree of distortion in the EOD waveform (Figure 1A, in red), to which one class of electroreceptors (B-cells) is uniquely tuned (von der Emde & Bleckmann, 1997). This mechanism makes prey and predators pop out from a background of nonliving objects. Strikingly, the range of detectable capacitances in different species of mormyrids corresponds to the range of capacitances that populate their niches (von der Emde, 1990).

Finally, mormyrids also possess a surprising ability to recognize three-dimensional shape by electrolocation, despite variation in object size, pose, and material (von der Emde, 2004; von der Emde et al., 2010; von der Emde & Fetz, 2007). The results suggest that shape recognition may be based on the configuration of object parts and might even exploit temporal deformations of the field in an electric version of shape-from-motion.

In sum, a host of object properties are uniquely specified by higher order ratios of four variables: the peak amplitude, maximum slope, diameter of the electric shadow, and the distortion of the EOD waveform. These variables are informative by virtue of the laws of electrodynamics in an aquatic niche, including the resistance and capacitance of meaningful classes of objects. Upon investigation, electrolocation turns out to be an ecologically well-posed problem.

### The Narwal’s Tusk

What is the function of the narwal’s tusk? The question has been a matter of scientific speculation for over 500 years, with hypotheses ranging from a weapon or a spear (despite the problem of consuming impaled prey), to a rudder, a spade, or an ice pick (Nweeia et al., 2009). More plausibly, it might be a secondary sex characteristic that plays a role in sexual selection or social dominance, akin to the stag’s rack. But recent research indicates that it’s a sense organ!

**Figure 2.**
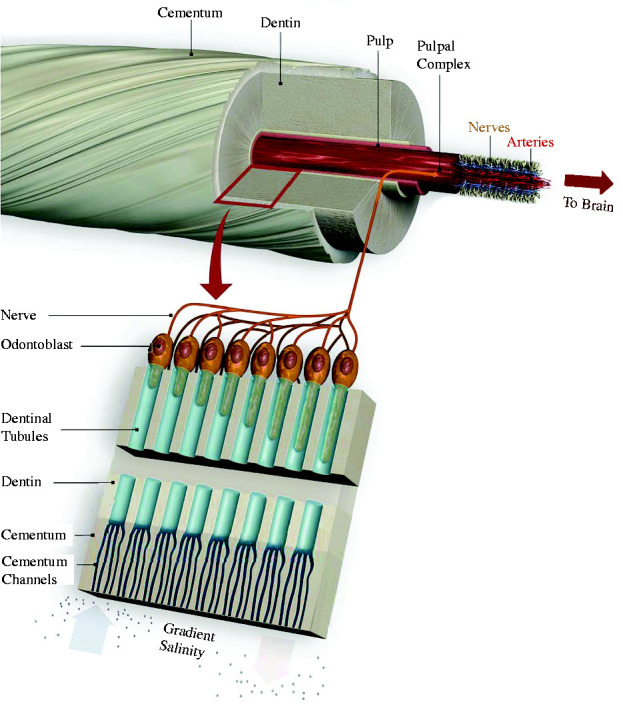
The narwal’s tusk contains dentin tubules that sense osmotic gradients (modified from Nweeia et al. (2014), with permission).

The male narwal’s tusk is a single erupted canine tooth, which forms a sinistral helix 2 to 3 m in length. A protective outer layer or *cementum* covers the hard interior *dentin*, which surrounds a pulp core containing vascular and nervous tissue (Figure 2; Grandfield et al., 2014; Nweeia et al., 2009). As in human teeth, hollow *dentin tubules* run radially from microchannels in the cementum to neural odontoblasts in the pulp. Unlike human teeth, however, these channels are not covered by an enamel layer but are open to the external ocean. The narwal’s dense network of fluid-filled tubules could thus conduct changes in temperature, pressure, or electrochemical gradients to the odontoblasts, the maxillary branch of the fifth cranial nerve, and thence to the brain (Nweeia et al., 2014).

Now consider the narwal’s ecological niche. The narwal is an arctic whale that hunts halibut in complete darkness using click echolocation, deep beneath the winter pack ice. It can dive to depths of 1,500 m for up to 25 minutes, reaching pressures greater than 150 atmospheres (Laidre et al., 2003). But at their wintering grounds, ice covers 90% or more of the water surface (Heide-Jørgensen & Dietz, 1995). As a mammal that must surface regularly to breathe, there is thus strong selective pressure to avoid getting trapped under rapidly forming and shifting sea ice.

When surface water freezes, the salinity of the water below the ice increases (National Snow and Ice Data Center, 2020). Thus, a narwal swimming up to surface ice encounters a salinity gradient in space (along the tusk) and time (as it moves up the gradient). A higher concentration of sodium and chloride ions in the seawater generates an outward osmotic flow in the dentin tubules, stimulating the odontoblasts (Figure 2, bottom); conversely, a lower concentration generates an inward osmotic flow (Nweeia et al., 2014).

To investigate this hypothesis, Nweeia et al. (2014) alternately injected salt and fresh water into a tube surrounding the tusks of six male narwals while recording an electrocardiogram. A higher salinity evoked a significant 15% increase in heart rate. This result is consistent with the hypothesis that salinity differentials provide effective information for an imminent threat.

Within the narwal’s arctic niche, salinity gradients thus specify a very relevant property: the penetrability of the surface. The laws of chemistry, together with the niche’s regularities, grant salinity the status of information for the *affordance* of penetrability. The dentin tubule system is nicely tuned to this information, which is advantageous for a mammal’s survival under the ice.^2^ According to Gibson’s perceptual theory, when the narwal’s tusk detects information (a salinity differential), the narwal perceives the specified environmental property (an impenetrable surface).

In sum, information is where you find it—in ecological niches. Research inspired by Gibson’s information hypothesis has found that this is also true for vision. Let us press on.

## Optic Flow: Steering Through Gaps

A goshawk flying through its dense woodland habitat deftly avoids trees and brush, threading its way through narrow gaps, all at break-neck speed. Video from a hawk head-cam during such maneuvers is astounding (BBC, 2009, min 1:43). Humans do this too, of course—only at lower speeds—any time we walk through a cluttered room or down a busy sidewalk. What information is used to guide locomotion, avoid obstacles, and steer through apertures or gaps? Gibson’s proposed solution was *optic flow* (see also Rogers, 2021).

**Figure 3.**
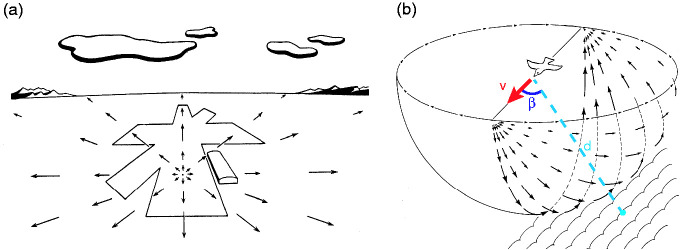
Optic flow represented as a velocity field. A: Radial pattern of outflow during a landing glide, from the pilot’s viewpoint. The focus of expansion corresponds to the airplane’s current heading direction. B: Velocity field from a third-person viewpoint, projected onto a sphere about the observer. *V* = observer’s speed and heading, *d* = distance to a point in the environment, β = visual angle between them. Vectors represent angular optical velocities ($\dot{\beta }$) of environmental points (from Gibson, 1950, 1979, permission pending).

Gibson discovered the optic flow pattern during the World War II, when he was leading research on pilot testing and training for the U.S. Army Air Force (Gibson, 1947).^3^ By mounting a camera in the nose of an airplane during glide landings and projecting the images on a screen, he and his colleagues traced out the radial pattern of velocity vectors that came to represent the optic flow field (Figure 3A). In particular, they found that the focus of expansion in the velocity field corresponded to the current direction of travel or *heading*. The magnitude of each vector could be calculated by the basic flow equation (Gibson et al., 1955; Nakayama & Loomis, 1974; see Longuet-Higgins & Prazdny, 1980, for the general form):

(1) $$\dot{\left|\beta \right|}=\frac{|V|\sin\beta }{d}$$

where *d* is the distance to a point in the environment, β is the visual angle between the point and the focus of expansion, |*V*| is the observer’s speed, and vector directions radiate from the focus (Figure 3B).

Gibson (1950, 1966) promptly criticized the velocity field as only a partial description of optic flow, for it failed to capture local deformations, texture accretion/deletion, and temporal derivatives such as acceleration. He defined optic flow more broadly in 1966:

**Optic flow:** The pattern of change in the optic array at a moving point of observation.

The optic flow pattern is a rich source of information about self-motion and spatial layout, available to both compound and chambered eyes. Potentially informative variables include the focus of expansion or radial outflow, the point of zero parallax, local expansion/contraction, differential motion, the acceleration field, and so on. The projection of optic flow onto a moving receptor surface, called *retinal flow*, is more complicated because it is affected by eye rotations (Gibson, 1950), but the visual system manages to extract information about self-motion from the retinal flow pattern nonetheless (for more, see Li et al., 2006; Warren, 2008).

What does this have to do with steering through gaps? One of Gibson’s most influential proposals is that optic flow is used to control locomotion. He initially suggested that steering is “a matter of *keeping the focus of expansion in the direction one must go*” (Gibson, 1950, p. 128), and he subsequently proposed a set of “formulae” (Gibson, 1958, p.186) or “rules” (Gibson, 1979, p. 232) for the control of specific locomotor behaviors. In particular, he hypothesized that steering is controlled by keeping the focus of radial outflow outside patches of the optic array that specify obstacles and inside patches that specify openings. Approach to a goal and pursuit of a target are controlled by local expansion, escape from a pursuer is controlled by local contraction, and following is controlled by both. Contrary to the premise of underdetermination, there are actually myriad variables that might be exploited for each of these tasks (see Warren, 1998), and testing them across species has become something of a cottage industry (Altshuler & Srinivasan, 2018; Pepping & Grealy, 2007; Serres & Ruffier, 2017; Srinivasan, 1998).

Srinivasan noticed that honeybees enter the hive by flying through the center of the entrance hole, leading him to suggest that they fly through gaps by balancing the rate of optic flow in the left and right eye. This is close to Gibson’s (1958, 1979) formula of symmetrically magnifying the local contour of the opening, but only in the horizontal dimension. Srini and his colleagues (Kirchner & Srinivasan, 1989; Srinivasan et al., 1991) tested the hypothesis by flying bees down a vertically striped corridor while manipulating the speed of the stripes on one wall. Just as predicted, the bees shifted away from the wall with faster motion to the balance point in the corridor where the left and right flow rates were equalized.

When Andrew Duchon and I (Duchon & Warren, 2002) asked people to walk down a virtual hallway on a treadmill and similarly manipulated the speed of motion on one wall, humans behaved just like honeybees, shifting to the predicted balance point in the hallway. We implemented the balance strategy in a mobile robot (Duchon & Warren, 1994; Duchon et al., 1998) and found that it makes an excellent obstacle-avoidance strategy: The robot veers away from obstacles because they generate a higher rate of optic flow in one hemifield. This is reminiscent of Gibson’s (1958) formula for steering around obstacles by using the skewed magnification of the obstacle’s contour.

Reality turns out to be more complex, of course. Subsequent work has revealed that honeybees can also follow one wall in a corridor, by holding its optical flow rate constant (Serres et al., 2008); the lateral positions of the bee and its goal determine whether the bee adopts a balancing or wall-following strategy. Because humans walk on the ground, we equalize not only the flow rates of the left and right walls in a hallway but also the splay angles of the left and right baseboards (Duchon & Warren, 2002) and the edges of a path (Beall & Loomis, 1996).

While these findings tell us how bees and humans travel down a corridor, they do not yet answer the question of steering through gaps. There are a number of alternative hypotheses (Warren, 1998). First, there is Gibson’s (1958, 1979) global flow hypothesis: shift the focus of outflow (or more generally, the heading specified by optic flow) onto the gap. Second, there is Gibson’s (1958) local expansion hypothesis: symmetrically magnify the contours of the gap (akin to balancing the left and right flow rates). Third, cancel the lateral motion of the gap, originally described by Llewellyn (1971, p. 246) as “target drift”. Fourth, steering might not be based on optic flow at all, but on positional information (Rushton et al., 1998): move in the egocentric direction of the gap, or center the gap at the midline and move forward. Such a strategy may be needed to walk toward a distant light in the dark, but optic flow might be useful under illumination.

**Figure 4.**
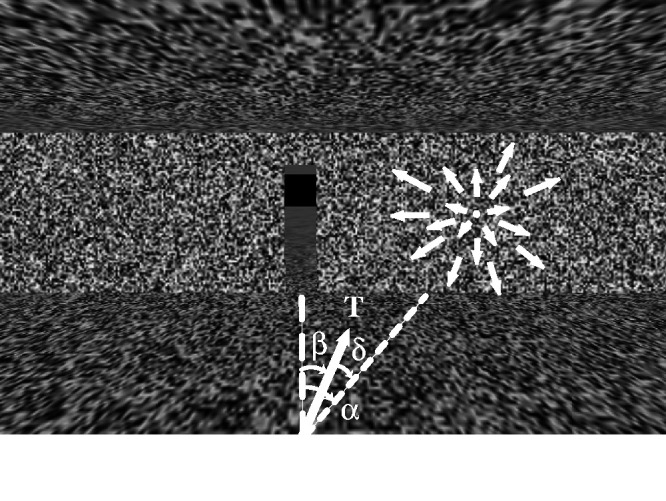
Walking to a gap in virtual reality, with displaced optic flow. *T* = walking direction, δ = displaced radial outflow, β = angle between walking direction and gap (from Warren et al., 2001, with permission).

We tested these hypotheses by using virtual reality to dissociate the optic flow pattern from the actual direction of walking (Warren et al., 2001). Participants viewed a virtual environment in a mobile head-mounted display, while we displaced the focus of expansion by δ = 10˚ left or right from their walking direction (T). In Figure 4, the participant begins walking with the focus of expansion to the right of the gap. If they steer by shifting the focus (or flow-specified heading) onto the gap, they would then be walking toward the left wall. Over time, however, they would trace out a straight path that intersects the gap. That would also be the case if the participant steered by canceling target drift, which is equivalent to shifting the focus of expansion onto the gap (it is the only fixed point in the flow field). On the other hand, if the participant steered by walking directly toward the gap’s current position, the focus of expansion would remain 10˚ to the right, so the gap would gradually drift leftward. Over time, they would trace out a curved path to the gap.

**Figure 5.**
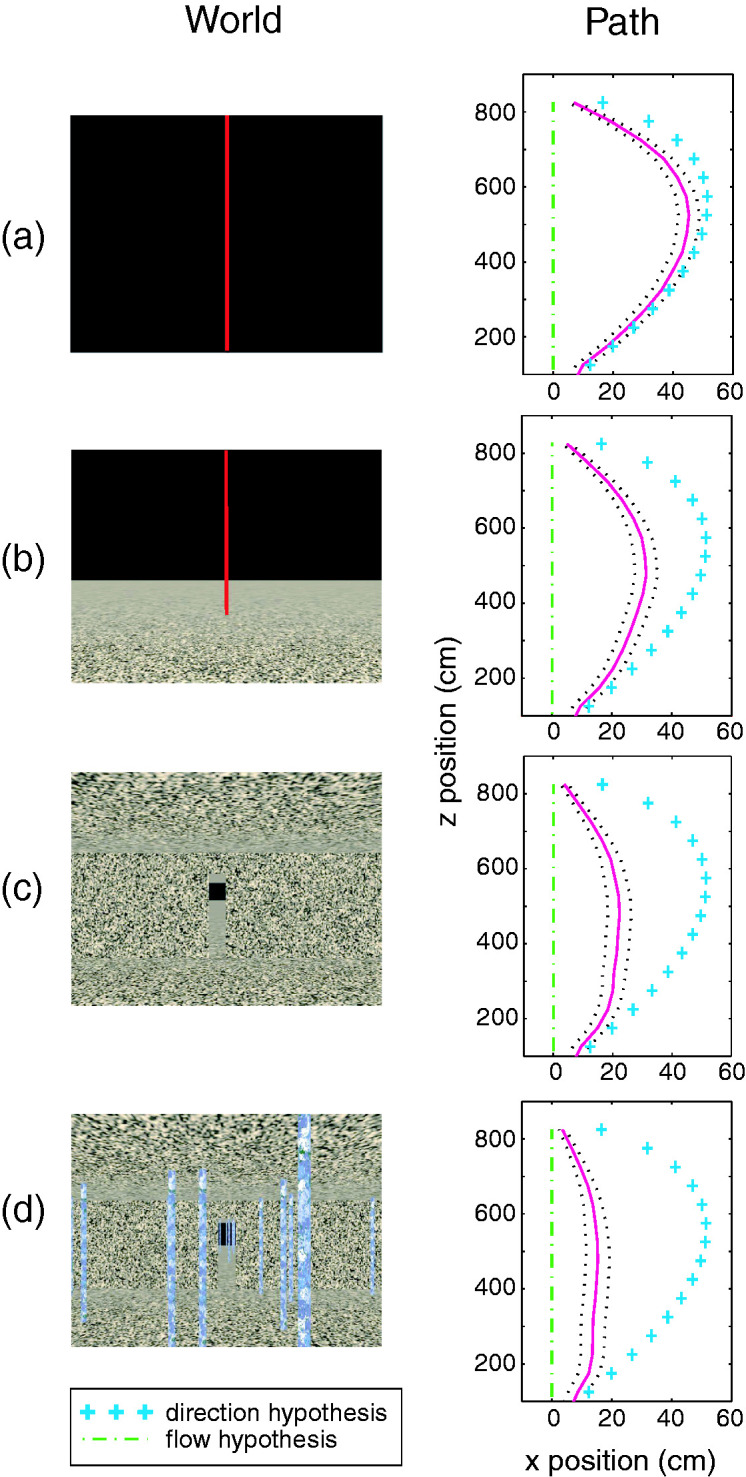
Virtual environment (left) and mean path (right, magenta ± *SEM*) in each condition, with predictions of the positional hypothesis (+) and the flow hypothesis (_._)(from Warren et al., 2001, with permission).

With only a single target in the dark, that is precisely what happened: Participants walked on a curved path to the target, as predicted by the positional hypothesis (Figure 5A). This also demonstrates that they did not cancel target drift, which predicts a straight path. However, as more textured surfaces were added in the display, the amount of optic flow increased, and the paths increasingly straightened out, as predicted by the flow hypothesis (Figure 5B to D). Paths were straightest when a forest of poles was present (Figure 5D), consistent with the use of differential motion: The differential motion field also forms a radial pattern, with the point of zero parallax in the heading direction.

The results indicate that humans rely on both optic flow and positional information to steer to a goal or a gap, with the former dominating as more flow is added to the display. This is a control strategy that is robust to variation in viewing conditions. Naturally, the debate has continued, with some results favoring the positional hypothesis (J. M. Harris & Bonas, 2002; Rogers & Allison, 1999; Saunders & Durgin, 2011) and others the optic flow hypothesis (Bruggeman et al., 2007; M. G. Harris & Carre, 2001; Turano et al., 2005; Wood et al., 2000). Recent research has confirmed that the relative contribution of optic flow and positional variables depends on the available flow (Li & Niehorster, 2014; Saunders, 2014).

## Gibson’s Affordance Hypothesis: Passable Gaps

Steering through a gap is not sufficient for successful behavior, however. To avoid bodily harm, the hawk, the human, and the bee must also be able to see whether the gap is large enough to fit through, that is, whether it is *passable*. Passability is a prime example of what Gibson (1979) called an *affordance* for behavior. He defined affordances as properties of the environment “taken with reference to” (p. 137) an animal’s body and action capabilities. Thus, a gap is passable if and only if its horizontal width is greater than the locomoting animal’s body width; otherwise, a change in behavior is called for. Affordances are everywhere: graspable objects, walkable surfaces, climbable slopes, throwable projectiles, catchable prey, edible food, habitable shelters, cutting or pounding tools, and so on. Indeed, Gibson proposed that an ecological niche *is* a set of affordances, which co-evolve with the action capabilities of the species.

To characterize an affordance, Gibson (1979, p. 127) said that environmental properties must be “measured *relative to the animal*.” Following principles of geometric and dynamic similitude (Schuring, 1977), affordances can be expressed as dimensionless ratios of environmental and animal variables (Warren, 1984). In dimensional analysis, such ratios are called *π-numbers* (Buckingham, 1914). At critical values of a π-number, the system’s behavior changes qualitatively, and because π-numbers are dimensionless, their critical values are scale-invariant. A good example is the Reynolds number, whose critical values capture the transition from laminar to turbulent fluid flow in systems of different scales.

Applying this way of thinking to the humble gap, passability may be characterized by a dimensionless π-number,

(2) $$\pi =\frac{G}{W}$$

where *G* is gap width, *W* is frontal body width, and a critical value $\pi_{c}$ expresses the boundary between passable and impassable gaps. Such body-scaled (geometric) or action-scaled (dynamic) ratios capture affordances that are invariant across individuals of different sizes. In principle, higher order affordances could be characterized by increasingly complex π-numbers.

**Figure 6.**
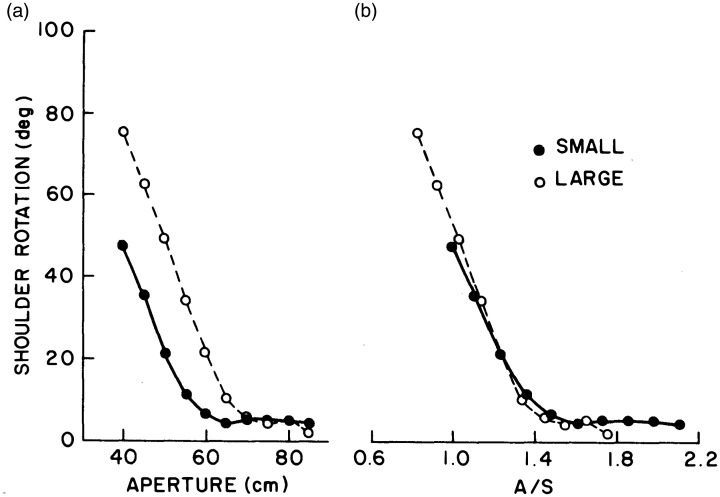
Mean maximum shoulder rotation when walking through an aperture as a function of (A) aperture width and (B) the ratio of aperture width to shoulder width, for small and large participants (from Warren & Whang, 1987, with permission).

To test the prediction that affordances are scale-invariant, the late Suzanne Whang and I (Warren & Whang, 1987, Exp. 1) asked large and small adults to “walk naturally” through gaps (apertures) of different widths. As the aperture got smaller, at some point participants began to rotate their shoulders and pass through sideways. This behavioral transition occurred at a narrow gap for small people and a wide gap for large people (Figure 6A) —but when shoulder rotation was replotted as a function of the ratio of aperture width to shoulder width *A/S* (equivalent to *G/W*) the two sets of data collapsed (Figure 6B): The onset of rotation occurred at the same critical value ($\pi_{c}=1.3$) and the groups behaved identically as they passed through the gap. The boundary between passable and impassable gaps thus falls at an aperture width that is 1.3 times shoulder width, regardless of body size, allowing a safety margin that correlates with body sway (Snapp-Childs & Bingham, 2009; Wilmut & Barnett, 2011). The affordance of passability is thus scale-invariant.

This brings us to what is perhaps Gibson’s (1979) most notorious claim—that affordances not only exist, but can be *perceived:*

**The Affordance Hypothesis:** An affordance is perceivable if there is higher order information, however complex, that specifies the relation between environmental properties and animal properties that constitutes it.

Note what the hypothesis does *not* say. It does not claim that all affordances can be perceived; that is an empirical question. Neither does it claim that all affordances are specified by information. Nor does it assert that affordances are perceived spontaneously, for the affordances of terrain, food items, and projectiles may be discovered by exploration, and the observer may become attuned to information through perceptual learning. Rather, the claim is that affordances are potentially perceivable if they are grounded in information specific to the relevant environment–animal relations^4^—if only we are clever enough and dogged enough to find it.

### Body-Scaled and Action-Scaled Information

Gibson (1979, p. 127) reasoned that, according to the Information Hypothesis, many environmental properties such as the layout and composition of surfaces are optically specified. If a behaviorally relevant complex of surface properties constitutes an affordance, then “to perceive them is to perceive what they afford.” Affordances are not merely combinations of neutral physical properties, however, for when considered in relation to an animal, the complex has “unity,” “value,” and “meaning” for behavior. He offered an example: A surface that is horizontal, flat, extended, rigid, and low, relative to the animal’s body size, weight, and leg length, might be specified by a higher order combination of optical variables. This “compound invariant” (p. 141) would thus specify the affordance of a walkable surface.

**Figure 7.**
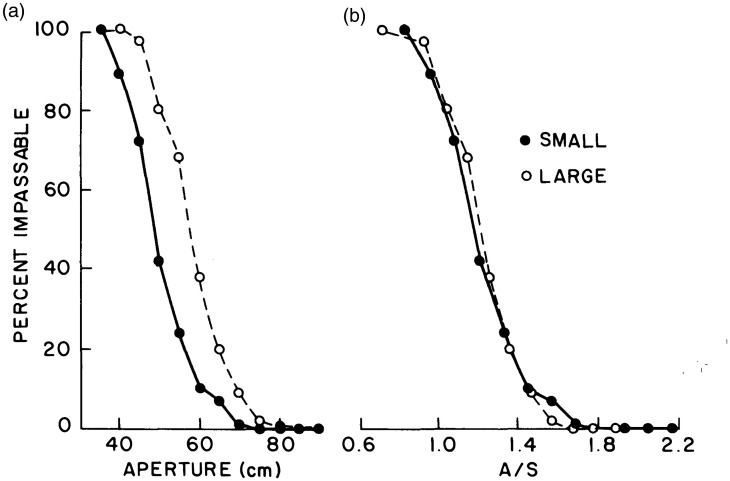
Mean percentage of “impassable” judgments with static monocular viewing as a function of (A) aperture width and (B) the ratio of aperture width to shoulder width, for small and large participants (from Warren & Whang, 1987, with permission).

To test empirically whether humans can perceive passable gaps, Suzanne and I asked large and small participants to judge whether they could “walk straight through” gaps of different widths without turning their shoulders (Warren & Whang, 1987, Exp. 2). In the static monocular condition, the gap was viewed through a reduction screen at a distance of 5 m, and in the moving binocular condition while walking from 7 m to 5 m. The results were the same in both conditions: The 50% threshold gap size was wider for large people than small people (Figure 7A), but when plotted as a function the body-scaled ratio *A/S*, the perceptual boundary fell at a critical value of 1.16 for both groups (Figure 7B). Slightly narrower gaps were judged to be passable than indicated by shoulder rotation when actually walking through them (Figure 6B), perhaps due to the slight difference in instructions. Nonetheless, the perception of passable gaps was scale-invariant, whether based on static or dynamic information.

Such evidence indicates that affordances can be judged successfully. It might be objected that, while the environment may be perceived, affordances are surely inferred based on prior knowledge of one’s body plan and motor abilities. In contrast, Gibson claimed that affordances are perceived *per se*, based on information about the relevant complex of environmental–animal relations. At the heart of this claim lies the notion of *body-scaled* or *action-scaled* information (Lee, 1980; Warren, 1984, 2007), the idea that visual information can specify the relation between environmental properties and the animal’s action system. Information can be scaled by calibrating optical variables to action variables through context-specific experience (Franchak et al., 2010; van Andel et al., 2017).

**Figure 8.**
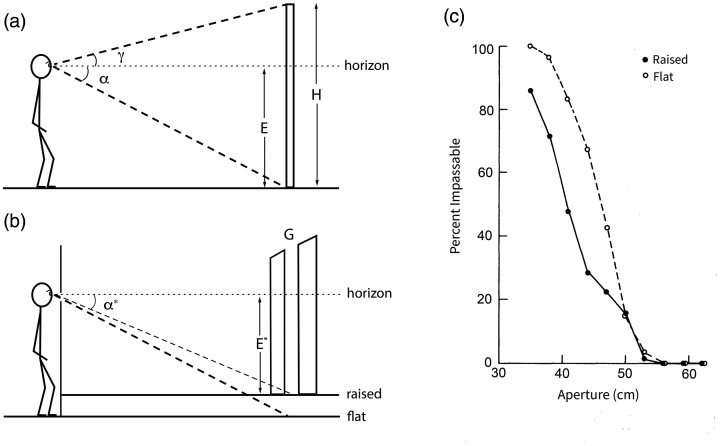
Eyeheight-scaled information for gap width. A: Definition of variables for the horizon ratio (Equation 3). B: Raising a false floor reduces the declination angle ($\alpha^{*}$) and increases the specified gap width (Equation 4). C: Mean percentage of “impassable” judgments: smaller gaps are judged as passable when the floor is raised (from Warren & Whang, 1987, with permission).

Pursuing the example of the gap, what is the body-scaled information for passability? Somehow an optical variable that incorporates a “length” scale for body size must be found. In terrestrial animals, this is given by standing eye height. Sedgwick (1986, 2021, also Gibson, 1979) showed that the height (*H*) of an object is specified as a ratio of the observer’s eye height (*E*) by the *horizon ratio* (Figure 8A):

(3) $$\frac{H}{E}\text{=}\frac{\tan\alpha +\tan\gamma }{\tan\alpha }$$

where α is the declination angle from the horizon^5^ to the base of the object on the ground plane, and γ is the visual angle between the horizon and the top of the object. The same is true for the frontal width of a gap (*G*), where β is half the horizontal visual angle of the gap:

(4) $$\frac{G}{E}\text{=}\frac{2t\mathrm{an}\beta }{\tan\alpha }$$

For a walking observer, eye height is also specified by the height of the focus of expansion on surrounding surfaces (Lee, 1980; Wu et al., 2005). Thus, consistent with our results, aperture width is specified as a ratio of eye height by static and dynamic^6^ information. Because shoulder width is about one-quarter of standing eye height, the perceptual boundary (*A/S* = 1.16) can also be expressed as a ratio of eye height (*A/E* = 0.29). In other words, once the horizon ratio is calibrated to shoulder width, a critical value of 0.29 specifies the boundary between passable and impassable gaps.

To test this body-scaled information, we manipulated the horizon ratio by raising a false floor behind the reduction screen (Figure 8B), unbeknownst to the observer (Warren & Whang, 1987, Exp. 3). This served to reduce the declination angle ($\alpha^{*}$) from the horizon to the bottom of the gap, so the horizon ratio specified a wider gap (Equation 4). The horizon ratio hypothesis thus predicts that narrower gaps should look passable. Specifically, if participants rely on a critical horizon ratio of 0.29, then raising the false floor should shift the perceptual boundary to a smaller aperture width. That is precisely what we observed (Figure 8C). Moreover, when the perceptual boundary was expressed as ratio of the effective eye height (E*) in the raised floor condition, the critical ratio was A/E* = 0.29, virtually identical to *A/E* = 0.28 in the flat floor condition—just as predicted.

The horizon ratio thus provides effective body-scaled information for passable gaps. If one manipulates the visual information—in this case the effective eye height—the perception of other eye height-scaled affordances should also shift. But if one manipulates the action system—such as changing body width by wearing a backpack—the information may need to be recalibrated to the new action capabilities (Franchak, 2017; Mark, 1987; Mark et al., 1990; Pan et al., 2014; van Andel et al., 2017).

Note that eye height-scaled information only works in terrestrial niches where animals walk and obstacles rest on the ground plane. The horizon ratio is informative about gap size by virtue of the laws of optics and gravity. Other body-scaled information must be available in aerial niches, where goshawks, budgerigars, and bumblebees successfully navigate through narrow gaps (Ravi et al., 2019; Schiffner et al., 2014).

### Bumblebee Affordances

**Figure 9.**
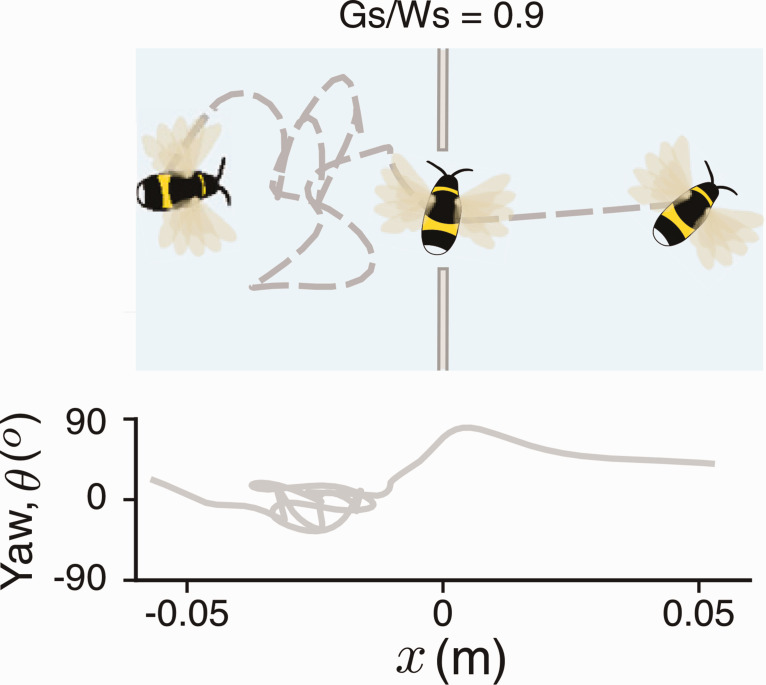
Top-down view of a bumblebee flying through a narrow gap. Note lateral “peering” movements in front of gap, and yaw angle up to 90˚ within gap (from Ravi et al., 2020a, with permission).

Not long ago I received a call from Sridhar Ravi, an aerospace engineer at the University of New South Wales, who was flying large and small bumblebees through gaps of various widths. He said that his bees were behaving just like our humans: As gap width (*G*) decreases, the bee begins to pivot about a vertical axis and fly through the gap *sideways* (Figure 9), analogous to humans rotating their shoulders. Because the bee’s body length is only half its wingspan (*W*), this yaw maneuver enables them to pass through much narrower gaps (Ravi et al., 2020a), as Sridhar’s slow-motion videos illustrate (Ravi et al., 2020b).

**Figure 10.**
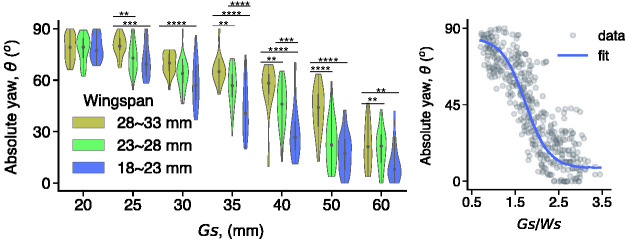
Yaw angle when flying through a gap. A: Yaw angle as a function of gap width for small, medium, and large bumblebees. B: Same data plotted as a function of the ratio of gap width to wingspan, with sigmoidal fit (*R*^2^ = .82; from Ravi et al., 2020a, with permission).

Importantly, the onset of yaw occurs at wider gaps for larger bees and narrower gaps for smaller bees (Figure 10A). Not only that, but when yaw angle is replotted as a function of π=G/W, the data from different-sized bees collapse and the yaw onset occurs at the same critical value, about $\pi_{c}=2$ (Figure 10B). At smaller ratios, the frequency of contact with the edges of the gap begins to go up, indicating that $\pi_{c}=2$ provides an appropriate safety margin. These results show that bumblebees perceive gap width relative to their own body size. A similar relationship has been observed in budgerigars, who fold their wings when gap width gets very close to their wingspan (Schiffner et al., 2014). Just like humans, bumblebees and budgerigars perceive the scale-invariant affordance of passability.

So what’s the body-scaled information? Because bees are aloft, eye height-scaled variables provide no information about size. And with the notable exception of the praying mantis (Nityananda et al., 2016), invertebrates do not possess stereopsis. Sridhar found a clue in their “peering” behavior: As a bee approaches a gap smaller than ∼2 wingspans, it starts to hover in front of the gap and oscillate from side to side, keeping the edges in its field of view (see Figure 9). As the gap narrows from ∼2 to <1 wingspans, the mean number of passes increases from 2 to 10, and the mean peering time grows from <1 second to 4 seconds (Ravi et al., 2020a). This strongly suggests that the bees are scanning the edges of narrower gaps to determine their width from optic flow.

The puzzle is how a “length” scale gets into the optic flow during free flight (Gibson described the same puzzle for pilots in 1947). A reference flight speed would help. For example, if it can be assumed that the speed of approach to a gap is always the same, gap width is specified by the visual angle of the gap and its rate of expansion (Schiffner et al., 2014). Unfortunately, bumblebees do not have a constant approach speed—but they might perceive gap width based on the lateral velocity of their peering movements.

A bumblebee produces lateral movements by rolling its body about the longitudinal axis while holding its head vertical, locked to the visual surround. It is safe to assume that the body roll angle relative to the head is specified by neck proprioception from mechanosensory hair fields, as in other insects (Preuss & Hengstenberg, 1992). Remarkably, for any hovering body from bees to helicopters, lateral acceleration (*a*) is directly proportional to roll angle (ρ) for small angles: a=gρ, where *g* is gravitational acceleration. Given that a peering movement accelerates from rest on each pass (Figure 9), it follows that the bee’s lateral velocity (*V*) is directly proportional to the elapsed time from the onset of each lateral movement^7^:

(5) V(t)=gρt

**Figure 11.**
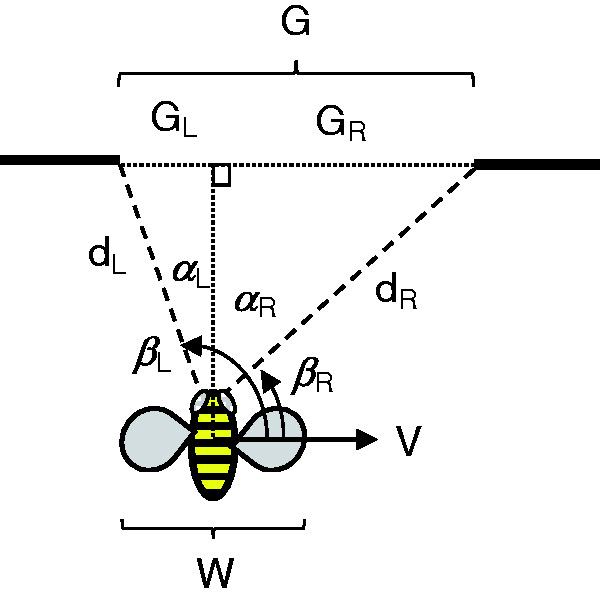
Optical information for gap width (see Equations 6 and 7; from Ravi et al., 2020b, with permission).

Rearranging the basic flow equation (Equation 1), the distance from the bee to the left and right edges of the gap (*d_L,_ d_R_*) is specified by the visual direction of the edge ($\beta_{L}$, $\beta_{R}$) and its optical velocity (${\dot{\beta }}_{L}$, ${\dot{\beta }}_{R}$; refer to Figure 11):

(6) $$d_{L}=\frac{V(t)\sin\beta_{L}}{{\dot{\beta }}_{L}},\;d_{R}=\frac{V(t)\sin\beta_{R}}{{\dot{\beta }}_{R}}$$

After a little trigonometry (see Ravi et al., 2020b), we find that gap width (*G*) is specified by a higher order relation between optic flow and neck proprioception:

(7) $$G=\;g\rho t\left(\frac{\sin\beta_{L}c\mathrm{os}\beta_{L}}{{\dot{\beta }}_{L}}+\frac{\sin\beta_{R}c\mathrm{os}\beta_{R}}{{\dot{\beta }}_{R}}\right)$$

It seems likely that this information is calibrated to a bumblebee’s wingspan by specific experience flying through gaps. For example, in each instar locusts recalibrate the information for gap sizes that their new bodies can step over, based on action-specific experience with walking across gaps (Ben-Nun et al., 2013). Similarly, humans calibrate the visual information for gap widths they can “squeeze” through, based on action-specific experience with squeezing (Franchak et al., 2010). When bumblebees fly through small gaps (<2 wingspans), their antennae, head, or legs frequently make contact with the edges of the gap, and wing collisions start to occur with narrower gaps (<1.5 wingspans). This might be a feature, not a bug: Mechanical contact provides feedback about gap passability that could be used to calibrate the optic flow (Equation 7). Thus, by virtue of the laws of optics and aerodynamics, and the bumblebee’s flight motor, wingspan-scaled optic flow is informative about the affordance of passable gaps.

In sum, birds do it, bees do it, even humans do it: perceive affordances based on body-scaled information, consistent with the affordance hypothesis.

## Conclusion

As Gibson (1979) foresaw 40 years ago, if we begin with cases of successful perceiving and acting, we often find informational variables that specify environmental properties and guide effective actions within the nomic constraints of an animal’s niche. Information is where you find it. The case studies I have reviewed here serve as existence proofs that information exists in wildly different energy arrays and is uniquely specific to behaviorally relevant properties for creatures great and small. Starting with the presumption that perception is an ill-posed problem leads us to abandon the search, sending vision science down the rabbit hole of prior knowledge. Gibson’s hypothesis that vision is ecologically well-posed holds out hope for a vision science grounded in natural law.

### Sidebar: Ecological Constraints or Prior Knowledge?

In one sense, perhaps, ecological constraints might be formulated as Bayesian priors. But in another sense, they are different animals: ecological constraints are facts of nature to which visual systems can adapt, whereas priors are internally represented *beliefs* about those facts, which are needed to make inferences about scene properties.

In their introduction to *Perception as Bayesian inference,* Knill et al. (1996) pointed out two possible interpretations of the Bayesian approach to vision. On the one hand, the approach provides a probabilistic framework for describing the constraints on scene structure (priors) and image formation (likelihoods) in order to specify the information content of images (posterior probabilities). It is thus a way to define the theoretical limit on performance for any vision system. In principle, one could go through the exercise of formalizing the physical and ecological constraints that render optical variables informative about properties of an econiche in this way. The *specificity* of Gibson information would correspond to posterior probabilities that are close to 1 (Nakayama & Shimojo, 1992). But making this move does not absolve the vision researcher of doing the scientific work of ecological physics and ecological optics: analyzing the natural constraints, the behaviorally relevant properties, and the actual information in particular niches.

As implied by the book’s title, however, the approach is commonly interpreted as a process model of visual perception. If perception is Bayesian inference, then the visual system combines probabilistic cues with internally represented knowledge (true beliefs) to make abductive inferences about the scene—that is, inferences from the image to beliefs about its best explanation. As Helmholtz understood, this prior knowledge must be of two kinds: knowledge about the external environment (priors) and knowledge about image formation (likelihoods; but see Feldman’s, 2013, caution). No sooner do Knill et al. (1996) invoke intentional concepts like knowledge, belief, and inference, though, before they back away from them, saying that this sort of prior knowledge may be no more than low-level filters or connection weights in a neural network. Palmer (1999, p. 83) executes a similar tactical retreat by saying that premises and assumptions may be merely patterns of neural connections, and “inference-like” processes are “somewhat metaphorical.” Indeed, the strong claims that synaptic weights in visual cortex represent knowledge or beliefs about the world, and neural networks make inferences, do not stand up to scrutiny (Orlandi, 2013, 2016). They are metaphorical. But metaphors guide the questions we ask, the experiments we do, and the theories we build.

Rather than internally representing external constraints, I would suggest we leave them in the environment where they belong. This would enable us to understand the visual system as *adapting to* the information they make available, in the course of evolution, development, and learning. The visual system need not internally represent facts about gravity or surface texture to enable successful perceiving, its neural networks just have to be tuned to the resulting patterns of stimulation. In van de Grind’s (1988) useful analogy, a fish need not know the laws of hydrodynamics in order to swim, its body and perceptual-motor loops merely need to be tuned to the properties of water. If one persists in calling such tunings “knowledge” and their activation “inference”, one persists in being metaphorical—and so does one’s theory.

## Notes

1 Although this is not Gibson’s exact wording, I believe it is his legacy. I derived this version from his hypothesis of psychophysical correspondence (Gibson, 1950, p. 8; 1959, p. 457, 465), adding the word “perceivable.”

2 There is a wrinkle to this story: male and female narwals have similar diving behavior, with comparable dive depths and frequencies in winter (Heide-Jørgensen & Dietz, 1995; Laidre et al., 2003), yet only 15% of females sport tusks (Nweeia et al., 2009). It is possible that other sensory systems can also detect surface ice (e.g., click echolocation); narwals live in mixed-sex pods, so social behavior could facilitate winter diving. Note that the male’s tusk may also be sensitive to pressure changes that are informative about dive depth, and chemical ion gradients that are informative about the presence of prey or mates in estrus.

3 This is actually a point of controversy (thanks to Dee Niehorster and Lothar Spillman for their comments). Mollon (1997) argues that Gibson took the concept of optic flow from G. C. Grindley. In a footnote in his tech report, Gibson (1947, p. 227) credits Grindley with describing the center of expansion in an unpublished, classified report to the British Flying Personnel Research Committee (Grindley, 1942). Grindley’s Appendix A includes a diagram of a radial vector field and the basic flow equation. In a thorough historical analysis, Niehorster (2021) finds that Gibson wrote a summary of Grindley's report in August, 1942; about the same time, he sketched his first diagrams of the flow field (similar to Figure 3a). However, whereas Grindley focused on the problem of estimating altitude from the optical velocity of points on the ground, Gibson grasped the significance of the whole flow pattern for perceiving the direction of locomotion. Contra Mollon, Gibson (1950) could not have cited Grindley's report because it was not declassified until 1972.

4 Compare Firestone’s (2013) critique of “ability-scaling.”

5 The horizon itself need not actually be visible, for it corresponds to the visually perceived eye-level, which is determined from the perspective of the ground plane and walls, the binocular disparity of the ground plane, and vestibular information for the inertial horizontal (Stoper & Cohen, 1986, 1989).

6 Fath and Fajen (2011) showed that there is also dynamic information for gap width scaled to body sway amplitude and stride length during walking.

7 This is also true for lateral body sway on each stride in human walking. Thus, Equation 7 might also explain Fath and Fajen’s (2011) data on walking through gaps without eye height information.
